# Optimization of the Extraction Conditions of Bioactive Compounds from *Ocimum basilicum* Leaves Using Ultrasound-Assisted Extraction via a Sonotrode

**DOI:** 10.3390/molecules28135286

**Published:** 2023-07-07

**Authors:** Carolina Aloisio, María del Carmen Razola-Díaz, María José Aznar-Ramos, Marcela R. Longhi, Alfonsina E. Andreatta, Vito Verardo

**Affiliations:** 1 Ingeniería de Procesos Sustentables, Facultad Regional San Francisco, Universidad Tecnológica Nacional, San Francisco 2400, Argentina; caloisio@facultad.sanfrancisco.utn.edu.ar (C.A.); aandreatta@facultad.sanfrancisco.utn.edu.ar (A.E.A.); 2 Unidad de Investigación y Desarrollo en Tecnología Farmacéutica (UNITEFA), CONICET, Córdoba 5000, Argentina; 3 Department of Nutrition and Food Science, Campus of Cartuja, University of Granada, 18071 Granada, Spain; carmenrazola@correo.ugr.es (M.d.C.R.-D.); mariajoseaznar@ugr.es (M.J.A.-R.); 4 Institute of Nutrition and Food Technology ‘José Matáix’, Biomedical Research Center, University of Granada, Avda del Conocimiento s/n., 18100 Granada, Spain; 5 Departamento de Ciencias Farmacéuticas, Facultad de Ciencias Químicas, Universidad Nacional de Córdoba, Ciudad Universitaria, Córdoba 5000, Argentina

**Keywords:** sweet basil (*Ocimum basilicum*), ultrasonic-assisted extraction via a sonotrode, Box–Behnken design, HPLC-ESI-TOF-MS

## Abstract

Sweet basil (*Ocimum basilicum)* leaves are rich in bioactive compounds that present therapeutic benefits for human health. Ultrasonic-assisted extraction (UAE) is frequently used to obtain phenolic compounds from plants/herbal sources. However, few works have developed multi-variable studies to find the optimal conditions to extract the maximum amount of compounds, especially when applied to UAE via a sonotrode. The purpose of this work was to perform a multi-variable study by employing a Box–Behnken design to collect the highest active compound content from *Ocimum basilicum* leaves. The efficacy of the design was endorsed by ANOVA. The studied parameters for UAE via a sonotrode were the ethanol/water ratio, amplitude, and time. The analyzed responses were the rosmarinic acid, the sum of phenolic acids, and the sum of phenolic compounds content. The optimal conditions were found to be 50% ethanol/water, 50% amplitude, and 5 min. Twenty bioactive compounds were identified by HPLC-ESI-TOF-MS when the extract was collected by applying the optimal conditions. *Ocimum basilicum* may be appreciated as a valuable source of important bioactive substances for pharmaceutical use.

## 1. Introduction

Extracts obtained from the leaves of *Ocimum basilicum* (sweet basil), belonging to the *Lamiaceae* family, present many active components, such as monoterpene hydrocarbons, oxygenated monoterpenes, oxygenated sesquiterpene hydrocarbons, sesquiterpenes, triterpenes, flavonoids, aromatic compounds, etc. These compounds have been reported to exhibit antibacterial, antifungal, antiviral, antiproliferative/anticancer, anti-inflammatory, antioxidant, antiulcer, insecticidal, and tissue-repair-promoting properties [1,2]. Among them, rosmarinic acid is a natural phenolic acid compound, structurally formed as an ester of caffeic acid and 3,4 dihydroxyphenyllactic that was first discovered in *Rosmarinus officinalis*, but it is present in most plants belonging to the *Lamiaceae* family, such as *Ocimum basilicum* [3,4]. It is recognized for its antioxidant qualities which allow it to neutralize free radicals, restricting oxidative effects in human cells and the proliferation of cancer cell lines [5]. Moreover, many other therapeutic effects have been reported, such as hepatoprotective [6,7], lung protective [8,9], and cardioprotective [10,11] responses, highly connected with antioxidant and anti-inflammatory activities [12]. In addition, Dahchour et al. described that this phenolic compound presents benefits for anxiety and depression [3]. One good use of biomass resources, which generates added value from technological developments, requires finding feasible, economic, and sustainable answers to problems of extraction, separation, purification, etc. To the present date, plant active substances are still traditionally extracted using hazardous and non-green solvents, such as methanol, ethyl acetate, acetone, dichloromethane, chloroform, hexane, etc. One of the disadvantages of processing with those solvents is that it requires complete elimination of them before being used as nutraceuticals or ingredients for the food, cosmetics, and pharmaceutical industries. Some natural extracts are also obtained through traditional techniques such as steam distillation, which must then be purified, and usually demand a high energy input. Due to the need for ecological processes and products, “green chemistry” promotes a large amount of research on the extraction and treatment of renewable raw materials to obtain natural products and molecules considered “building blocks” for fine chemistry through the development of safer, more sustainable industrial processes with less environmental impact. Thus, emerging pressurized fluid technologies use green and environmentally friendly solvents, as well as microwave-assisted extraction and ultrasound-assisted extraction [13].

Several methods have been reported for the extraction of phytochemicals from the leaves of *Ocimum basilicum*. Most methods are meant for the extraction of active substances from this plant by using either organic solvents [14,15,16], hydro-distillation [16,17,18], or microwave-assisted extraction with or without solvents [19,20,21]. Recently, ultrasound-assisted extraction (UAE) has gained attention. Ultrasonic baths and sonotrode equipment are two common instruments used for this purpose. Recently, a few works have reported the use of bath ultrasound for the extraction of active compounds from *Ocimum basilicum* [22,23,24]. Ultrasound baths impart a cheap and simple way to deliver ultrasound energy and allow for the use of a large assortment of solvents as extractants. However, the reproducibility is low, and the irradiation power is lower. Focused ultrasound systems overcome those problems [25]. Ultrasonic sonotrodes focus their energy on a particular sample zone, imparting more efficient cavitation in the fluid, which is an advantage over ultrasonic baths. Moreover, the higher intensity imparted by the ultrasonic probe system produces a much higher force in comparison to the ultrasonic bath (up to 100 times greater) [26]. Hence, it can increase the extraction rate from the plant material. Other advantages of UAE via a sonotrode are reduced extraction time, which allows for the use of green solvents, reduced energy consumption, and a safe extraction procedure, among others [27].

Although UAE has been previously reported in a few works for the bioactive compounds of *Ocimum basilicum* leaves, the studies have some limitations. For instance, Da s et al. studied different time conditions [22], Hashemi et al. studied different temperature values [24], and Ahmadi et al. varied both [22,24,28]. However, only Hashemi et al. used UAE via a sonotrode, and none of them identified the compounds via high-performance liquid chromatography with an electrospray ionization (ESI) source operating in negative mode and a time-of-flight (TOF) mass detector (ESI) (HPLC-ESI-TOF-MS) or developed a multi-variable design to optimize the extraction conditions [23]. From our knowledge, the unique work that describes a multi-variable study for the extraction of basil was the one reported by Soares et al. using an ultrasonic bath, but they did not take into account individual compounds [29].

The aim of this work was to develop a multi-variable study using a Box–Behnken design to obtain the highest active compound content from *Ocimum basilicum* leaves. Optimized factors of UAE via a sonotrode, such as ethanol/water composition, extraction time, and amplitude, were established. The identification and quantification of bioactive substances in the extracts were performed by HPLC-ESI-TOF-MS.

## 2. Results and Discussion

### 2.1. Identification of Polar and Other Compounds in Ocimum basilicum Extracts by HPLC-ESI-TOF-MS

**Figure 1:**
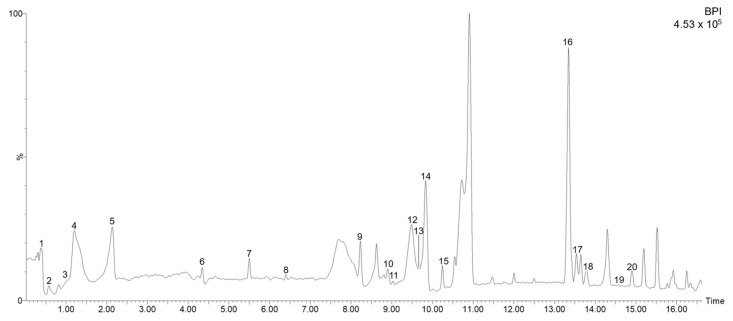
Base peak chromatogram of the compounds identified in the basil leaf. Numbers 1–20 correspond to the peaks in Table 1.

*Ocimum basilicum* leaf extracts obtained by UAE were analyzed by HPLC coupled to MS with a TOF analyzer. Active substances were identified by rendering their mass spectra, bearing in mind the data reported in the literature and, when available, by co-elution with commercial standards and using several databases. A total of 20 compounds were detected in the extracts collected from *Ocimum basilicum* leaves, of which 7 have been identified in basil leaves for the first time. All the identified compounds are shown in Table 1 with their retention time, experimental and calculated *m/z*, error (ppm) and score (%), main *m/z* in the source fragments, and molecular formulae. In Figure 1, a representative chromatogram of the basil leaf extracts analyzed by HPLC-ESI-TOF-MS is shown, whereby the numbers shown correspond to the peaks of Table 1.

Corresponding to peak 1 at 0.42 min with the *m/z* 133 and the molecular formula C_4_H_6_O_5,_ the compound was identified as malic acid, which has been found previously in foods such as unripe apples, grapes, watermelons, cherries, in vegetables such as carrots and broccoli [30], and in *Bryophyllum* [31] and *Raphanus sativus* [32] leaves. It is well known that malic acid in leaves is naturally involved in the regulation of stomatal opening and closing [33]. In addition, the malic acid derivative isopropylmalic acid isomers a and b were identified at 1.19 and 2.15 min (peak 4 and 5, respectively) with the *m/z* 175 and *m/z* in the source fragment 133, which corresponds to malic acid. It is an intermediate product in the production of the amino acid leucine previously described in several herbs and spices including sweet basil leaves [34]. The organic acid quinic acid isomers a and b were detected at 0.57 and 1.09 min (peaks 2 and 3, respectively) with the molecular formula C_7_H_12_O_6_. At 4.3 min (peak 6) with the *m/z* 179 and the molecular formula C_9_H_8_O_4_, the compound was identified as caffeic acid. Chicoric acid isomers a and b were detected at 8.29 and 9.51 (peaks 9 and 12, respectively), presenting the *m/z* 473. Corresponding to peaks 10 and 13 with a molecular formula of C_18_H_16_O_8_, the compounds were identified as rosmarinic acid isomers a and b, respectively. These compounds have previously been described in basil leaves by other authors [35,36,37]. Corresponding to peak 11 at 9.04 min and with the *m/z* 609 with an ion fragment at *m/z* 300, the compound was identified as quercetin rutinoside [38]. Peaks 17 and 18 with the *m/z* 313 correspond to cirsimaritin isomers a and b, respectively. This triterpenic acid has previously been found in basil leaves [39].

Otherwise, some compounds have been identified here for the first time. With a molecular formula of C_27_H_30_O_15_ (peak 15) at 10.21 min, the compound was identified as luteolin hexoside deoxyhexoside in agreement with its *m/z* in the source fragment 285 according to Mekky et al., who previously described it in sesame oil [40]. At 9.78 min (peak 14) with the *m/z* 717 and the predicted molecular formula of C_36_H_30_O_16_, the detected compound was proposed to be salvianolic acid as previously described in basil [38]. In the same way, isosalvianolic acid was identified at 14.92 min (peak 20). Peak 7 at 5.43 min with the *m/z* 387 and its source *m/z* fragments 207 and 179 was identified as the compound named tuberonic acid glucoside, also known as 12-hydroxyjasmonic acid glucoside, which was first identified in potato [41] and has also been found in other matrices such as lemon verbena [42] and thyme leaf [43]. The compound sesaminol triglucoside (PubChem CID:101394490) was identified at 6.33 min (peak 8) with the *m/z* 855 according to its source fragments 179 and 161. It has previously been described in sesame [44]. Peak 16, with the *m/z* 803 and its source *m/z* fragments 503, 357, 315, and 161, was identified as stevioside, in agreement with the Foodb database (FooDB ID: FDB013538). It has previously been described in stevia leaves [45], and this is the first time it has been described in basil leaves. Peak 19 at 14.74 min at *m/z* 345 presented a molecular formula of C_20_H_26_O_5_ with an ion fragment at *m/z* 283 and was identified as rosmanol. This compound has previously been identified and quantified in rosemary and sage [38].

### 2.2. Fitting the Model

The Box–Behnken experimental design established for the optimization of the conditions for UAE via a sonotrode is presented in Table 2. The evaluated independent factors were ethanol/water (*v*/*v*) (X_1_), amplitude (%) (X_2_), and time (min) (X_3_) at three levels, and the response variables to study were the sum of rosmarinic acid, the sum of phenolic acids, and the sum of phenolic compounds in order to maximize them.

As can be seen from the results, the sum of rosmarinic acid ranged between 8.26 and 420.13 µg/g d.w., the sum of phenolic acids ranged between 8.26 and 1458.90 µg/g d.w., and the sum of phenolic compounds ranged from 8.44 to 1559.45 µg/g d.w. In all cases, the minimum values corresponded to run 6 when using 100% ethanol, an amplitude of 20%, and 25 min; meanwhile, the maximum values were at the intermedium conditions (60% ethanol, 60% amplitude, and 25 min).

The experimental results were analyzed by adjusting them to a second-order polynomial regression (Equation (1)). The regression coefficients and analysis of variance (ANOVA) of the model and the results for the response variables for *Ocimum basilicum* are exhibited in Table 3.

The analysis of the model was performed for the three response variables. According to previous reports, the level of significance was α < 0.05 in order to enhance the number of meaningful variables [46,47]. The constants (β_0_) were significant for all of the responses (*p* < 0.05). For all of the response variables, the independent factors ethanol/water (X_1_) and amplitude (X_2_) showed significant effects in their linear (β_1_ and β_2_, respectively) and quadratic forms (β_11_ and β_22_, respectively) (*p* < 0.05). Moreover, the effect of all the crossed terms β_12_, β_13,_ and β_23_ were significant for the response variables studied. In the case of the sum of phenolic compounds, a significative effect of the que quadratic regression coefficient of time (X_3_) was also found (β_33_).

An analysis of variance (ANOVA) was performed after discarding the non-significant terms at α > 0.05. The models showed high determination coefficients between the independent factors and response variables (R^2^ > 0.9). Le Man et al. (2010) stated that a model is acceptable when R^2^ > 0.75 [48]. In addition, the *p*-values of the lack-of-fits were non-significant (*p* > 0.05). This parameter was used to verify the adequacy of the model, indicating that the model fits properly (Table 2). Moreover, the *p*-values of the models were lower than 0.05 for all of the models, making them statistically acceptable.

### 2.3. Establishment of Optimal Conditions

**Figure 2:**
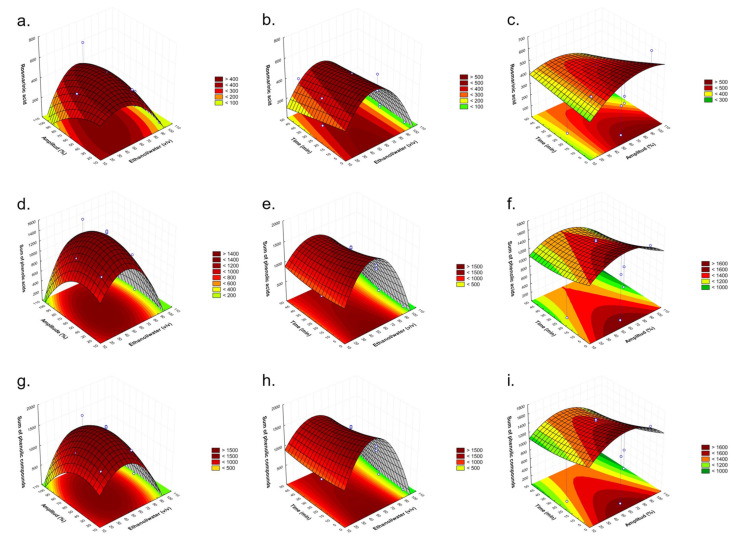
Response surface graphs (**a**–**i**) showing the combined effects of the independent factors: ethanol/water (*v*/*v*), time (min), and amplitude (%) for the responses of rosmarinic acid (**a**–**c**), the sum of phenolic acids (**d**–**f**), and the sum of phenolic compounds (**g**–**i**) expressed as µg/g d.w.

The three-dimensional plots showing the effects of ethanol/water % (*v*/*v*) (X_1_) with amplitude (%) (X_2_) (Figure 2a,d,g), ethanol/water % (*v*/*v*) (X_1_) with time (min) (X_3_) (Figure 2b,e,h), and amplitude (%) (X_2_) with time (min) (X_3_) (Figure 2c,f,i) on the rosmarinic acid, the sum of phenolic acids, and the sum of phenolic compounds content are presented in Figure 2.

In Figure 2a,d,g, the maximum rosmarinic acid, the sum of phenolic acids, and the sum of phenolic compounds content, respectively, were obtained with 40–50% ethanol/water and almost the whole range of amplitudes. In Figure 2b,e,h, the higher values were observed at 50% ethanol/water and for the whole range of time used for the three groups of compounds studied. And in Figure 2c,f,i, the maximum amount of compounds was obtained at approximately 50–100% amplitude and up to 10 min.

At 100% ethanol, when increasing the time, Tungmunnithum et al. [49] had the same tendency of increasing rosmarinic acid in *Plectranthus scutellarioides* L. leaves, as shown here (Table 2, runs 2 and 4).

In this work, we found that higher rosmarinic acid content was observed when increasing the power but reducing the time (Table 2, runs 9, 10, 11, and 2). This means that at a higher amplitude, time plays a more crucial role in increasing or reducing the rosmarinic acid content. Caleja et al. [50] in *Melissa officinalis* L. only saw differences in the content of rosmarinic acid, at fixed ultrasound power, with time at intermedium ethanol percentage; meanwhile, at extreme times, it showed no effect, and the same was true in this work (Figure 2b). Therefore, in contrast, when using the intermedium value of ethanol/water, higher extraction of rosmarinic acid is achieved with this solvent, maintaining the amplitude at the lowest or highest level but increasing the time, resulting in decreasing the content (Figure 2c).

The maximum values found under the medium amplitude condition could be justified by the higher temperature achieved in the process due to the effect of ultrasound, which can deteriorate the compounds. This effect also occurs when described in microwave extractions when high temperatures are used [51]. The higher ultrasound amplitude causes an increment in the temperature and pressure in the system [52,53]. Yang et al. also reported that extreme ultrasound forces may generate degradation of the substances due to the high temperature and pressure [54]. This finding is in agreement with a study conducted by Lanjekar et al. on poly-phenolic antioxidant extraction from waste mango peel [55] and the work published by Zheng et al. about phenolic compounds’ extraction from foxtail millet bran using UAE [56].

In addition, high time values may result in greater degradation of the target compounds or degradation of the matrix due to higher exposition to ultrasound forces, indeed when higher amplitude values are applied [51]. Moreover, the increment in the extraction time enhances the solution concentration, reducing the cellular osmotic pressure [57]. The result is in agreement with the results reported by Junchi et al., who extracted polysaccharides from *Ginkgo biloba* leaves [58]. On the other hand, an ethanol concentration higher than 60% did not increase the extraction rate, which may be caused by the change in the polarity of the solvent, which generates a negative effect on the extraction yield [59,60]. Similar responses have been described with other plant materials such as *Myrtus communis* [61] and *Solanum torvum Sw* [62] leaves.

### 2.4. Confirmation of the Optimal Extraction Parameters by a Sonotrode in Ocimum basilicum Leaves

After the optimal conditions were established by the response surface three-dimensional plots, the accuracy of the mathematical model was predicted, which constituted the final step of the RSM. The optimal conditions to obtain the highest rosmarinic acid amount, the sum of phenolic acids, and the sum of phenolic compounds from *Ocimum basilicum* and the predicted and obtained values of each response are presented in Table 4.

Briefly, the optimal conditions established were 50% ethanol/water (*v*/*v*), 50% amplitude, and 5 min. These values were found near the central point of ethanol/water and the amplitude. This performance is comparable with previous reports by our research group about the extraction of anthraquinones from *Heterophyllaea pustulata* [50] and by other authors for the UAE of antioxidant compounds from *Ficaria kochii* [63]. On the other hand, different behavior was observed for the time, which represented the minimum used value. These results may be explained by the fact that this ultrasound model decreases process times and due to the higher degradation of the compounds of interest or degradation of the matrix due to higher exposition to ultrasound forces, as mentioned in the above section.

The obtained predictable values under the optimal conditions were 529.97 ± 59.37 µg/g d.w. for rosmarinic acid content, 1657.15 ± 86.09 µg/g d.w. for the sum of phenolic acids, and 1749.04 ± 80.91 µg/g d.w. for the sum of phenolic compounds. Good agreement between the experimental and predicted values for all of the responses was found with a coefficient of variation lower than 2% in all cases. A few works have investigated different aspects of basil composition and extraction methods. Hashemi et al. reported the highest rosmarinic acid content and total phenolics (determined using the Folin–Ciocalteu method) for samples treated at 40% amplitude and 45 °C, reaching a maximum value of 156 mg/g [24]. The study highlighted the significant influence of temperature on the rate of rosmarinic acid extraction. Ahmadi et al. determined the optimum conditions for basil extraction, which were 40 min at 62.82 °C. The total phenolic compounds, measured using the Folin–Ciocalteu method, were reported to be 3922.485 mg/mL [64]. Soares et al. investigated the total phenolic content of basil in extra-virgin olive oil through the Folin–Ciocalteu method, which was reported as 135.94 mg GAE/kg. The results from these works indicated a higher concentration of phenolic compounds compared to our study, which may be explained by the fact that they determined the Folin–Ciocalteu reacting substances; meanwhile, in this work, the specific phenolic compounds were determined by HPLC-ESI-TOF-MS.

On the other hand, the other authors only quantified basil essential oil compounds, such as Silva et al., who measured linalool and estragole in basil, and Rajabi et al., who measured trans-anethole, estragole, and para-anisaldehyde in basil [23]. Additionally, Soares et al. evaluated different compounds such as alpha-thujene, beta-pinene, eucalyptol, beta-ocimene, camphor, linalool, eugenol, alpha-pinene, camphene, sabinene, myrcene, and limonene [29]. These compounds differ from the ones we evaluated in our study, preventing a direct numerical comparison.

Overall, comparing the numerical data from our work with the studies previously mentioned provides insights into the variations in basil composition and extraction methods. These differences highlight the importance of considering multiple studies to obtain a comprehensive understanding of the chemical profile of basil.

## 3. Materials and Methods

### 3.1. Samples and Chemicals

Fresh leaves of *Ocimum basilicum* were commercially acquired from a Spanish supermarket in June of 2022. The fresh leaves were air dried, protected from sunlight, and kept at an ambient temperature, and they were then micronized by employing a knife mill provided by IKA Werke GmbH & Co. KG (Staufen, Germany) and sieved to 0.2 mm. Then, the samples were stored in a freezer at −35 °C before analysis. HPLC-grade acetonitrile and acetone were supplied by Merck KGaA (Darmstadt, Germany), and purified water was obtained utilizing a Milli-Q system (Millipore, Bedford, MA, USA). Caffeic acid, quercetin, and rutin were provided by Sigma-Aldrich (St. Louis, MO, USA).

### 3.2. Experimental Design

In order to optimize the experimental conditions to collect the maximum amount of plant active substances in *Ocimum basilicum* leaves, a Box–Behnken design with three variables was performed. The three independent variables involved in this work were the ethanol/water ratio (X_1_), the amplitude of the ultrasound via sonotrode equipment (X_2_), and the extraction time (X_3_), with three values for each one. The response variables (Y) were the rosmarinic acid amount, the sum of phenolic acids, and the sum of phenolic compounds determined by HPLC-ESI-TOF-MS (Table 2). The percentages of ethanol/water were 20, 60, and 100% (*v*/*v*), and the extraction times were 5, 25, and 45 min. These conditions were selected considering previous reports on the extraction of rosmarinic-acid-related compounds from *Ocimum basilicum* and other plants [30,64,65,66] and *Ocimum basilicum* leaves [23,25]. Furthermore, the amplitudes of the sonotrode ultrasound equipment were 20, 60, and 100 (*%*), where the minor level was selected considering the preceding work conducted in our laboratory regarding the extraction of phenolic substances from orange by-products using UAE via a sonotrode [67].

In order to obtain the highest influence of unexplained variability in the responses found, caused by extraneous aspects, the experiments were randomized. In this sense, 15 sequences, along with the midpoint in triplicate, were tested (Table 1). The most suitable implementation for modeling is response surface methodology (RSM). In this approach, the second-order polynomial equation presented below (Equation (1)) is commonly used to study the interrelation between the independent and response variables [65]. The assay layout and evaluation of the most favorable UAE via the sonotrode settings in regard to the greater amount of active compounds were performed employing Statistica 7.0 software (StatSoft, Tulsa, OK, USA).

(1) $$Y=\beta_{0\;\;}+\sum_{i=1}^{3}\beta_{i\;\;}X_{i\;\;}+\sum_{i=1}^{3}\beta_{ii\;}X_{i}^{2}\;+\sum_{i=1}^{3}\sum_{j=i+1}^{3}\beta_{ij\;\;}X_{i\;\;}X_{j\;\;}{}_{\;\;}$$

where *X_i_* and *X_j_* are the independent variables, *Y* is the response variable, and *β_0_*, *β_i_*, *β_ii_*, and *β_ij_* are the constant, linear, quadratic, and cross-product coefficients, respectively [66].

### 3.3. Ultrasound-Assisted Extraction via a Sonotrode Applied to Ocimum basilicum Leaves

The procedure was performed by incorporating 0.25 g of *Ocimum basilicum* leaves with 10 mL of the chosen solvent. The ethanol used was absolute (>99% purity). A UP400St ultrasonic processor (Hielscher, Germany) with a working frequency of 24kHz and the sonotrode S24d3 were used. The ethanol/water ratio, US amplitude, and treatment time were adjusted in agreement with the assay layout. Subsequently, the suspensions were centrifugated at 6000 rpm for 10 min, and the supernatant was placed in a rotor evaporator and resuspended in 1 mL of methanol/water 50/50 (*v*/*v*). Finally, the samples were filtered with 0.2 μm nylon syringe membranes and kept at −18 °C until analysis.

### 3.4. Determination of Polar and Other Compounds in Ocimum basilicum Leaf Extracts by HPLC-ESI-TOF-MSs

*Ocimum basilicum* leaf extracts collected when UAE via a sonotrode was applied with different parameters was evaluated by an ACQUITY Ultra Performance LC system (Waters Corporation, Milford, MA, USA) associated with an electrospray ionization (ESI) source operating in negative mode and a time-of-flight (TOF) mass detector (Waters Corporation, Milford, MA, USA). All measurements were carried out in triplicate. The active substances were partitioned on an BEH Shield RP18 column (1.7 μm, 2.1 mm × 100 mm; Waters Corporation, Milford, MA, USA) at 40 °C using a gradient previously stated by Verni et al. 2020 [68] and using water containing 1% acetic acid as mobile phase A and acetonitrile as mobile phase B. The data were elaborated using MassLynx 4.1 software (Waters Corporation, Milford, MA, USA).

## 4. Conclusions

A Box–Behnken experimental design of 15 experiments was successfully performed to establish the optimal conditions of UAE via a sonotrode for *Ocimum basilicum* leaves to achieve extracts with high content in phenolic compounds. The extracts were analyzed by HPLC-ESI-TOF-MS, and a total of 20 compounds were identified, from which seven are described here for the first time in basil leaves. The analysis of variance showed a high correlation between the independent factors and response variables with determination coefficients (R^2^) and *p*-model values indicating that the model fits properly. The optimal conditions were established by RSM for the three responses studied (rosmarinic acid, the sum of phenolic acids, and the sum of phenolic compounds), and 50% ethanol/water, 50% amplitude, and 5 min were selected. Under these optimal conditions, the response variables were obtained experimentally and compared with the model predictions, and the validation of the model was confirmed statistically. In conclusion, *Ocimum basilicum* should be recognized as an important source of bioactive compounds, especially rosmarinic acid, with relevance for their therapeutic employment.

## Figures and Tables

**Table 1:** Identified compounds in *Ocimum basilicum* leaf extracts by HPLC-ESI-TOF-MS.

Peak	Time (min)	Experimental *m/z*	Calculated *m/z*	Error (ppm)	Score (%)	Molecular Formula	*m/z* in the Source Fragments	Compound Name
1	0.42	133.0139	133.0137	1.5	100	C_4_H_6_O_5_	-	Malic acid
2	0.57	191.0551	191.0556	−2.6	98.91	C_7_H_12_O_6_	111.0082	Quinic acid isomer a
3	1.09	191.0549	191.0556	−3.7	100	C_7_H_12_O_6_	133.0282	Quinic acid isomer b
4	1.19	175.0599	175.0606	−4.0	99.95	C_7_H_12_O_5_	133.0271	Isopropylmalic acid isomer a
5	2.15	175.0605	175.0606	−0.6	100	C_7_H_12_O_5_	133.0293	Isopropylmalic acid isomer b
6	4.30	179.0340	179.0344	−2.2	100	C_9_H_8_O_4_	133.0287	Caffeic acid
7	5.43	387.1653	387.1655	−0.5	100	C_18_H_28_O_9_	207.1017; 179.0712	Tuberonic acid glucoside
8	6.33	855.2519	855.2559	−4.7	95.08	C_38_H_48_O_22_	179.0699; 161.0605	Sesaminol triglucoside
9	8.29	473.0705	473.0720	−3.2	99.95	C_22_H_18_O_12_	293.0287	Chicoric acid isomer a
10	8.87	359.0768	359.0767	0.3	100	C_18_H_16_O_8_	133.0292	Rosmarinic acid isomer a
11	9.04	609.1469	609.1456	2.1	100	C_27_H_30_O_16_	300.0279	Quercetin-rutinoside
12	9.51	473.0740	473.0720	4.2	99.17	C_22_H_18_O_12_	293.0308	Chicoric acid isomer b
13	9.74	359.0778	359.0767	3.1	100	C_18_H_16_O_8_	133.0289	Rosmarinic acid isomer b
14	9.78	717.1451	717.1456	−0.7	97.38	C_36_H_30_O_16_	161.0246	Salvianolic acid
15	10.21	593.1498	593.1506	−1.3	99.96	C_27_H_30_O_15_	285.0395	Luteolin hexoside deoxyhexoside
16	13.38	803.3701	803.3701	0.0	100	C_38_H_60_O_18_	503.3382; 357.1906; 315.1796; 161.0463	Stevioside
17	13.58	313.0707	313.0712	−1.6	100	C_17_H_14_O_6_	161.0235	Cirsimaritin isomer a
18	13.82	313.0702	313.0712	−3.2	100	C_17_H_14_O_6_	161.0233	Cirsimaritin isomer b
19	14.74	345.1696	345.1702	−1.7	100	C_20_H_26_O_5_	283.1692	Rosmanol
20	14.92	491.0983	491.0978	1.0	100	C_26_H_20_O_10_	179.0345	Isosalvianolic acid

**Table 2:** Experimental Box–Behnken design of the conditions of extraction (independent factors) and the experimental results (response variables) obtained, expressed with the average ± standard deviation.

	Independent Factors			Response Variables		
	X_1_	X_2_	X_3_	Y_1_	Y_2_	Y_3_
Run	Ethanol/ Water (*v*/*v*)	Amplitude (%)	Time (min)	Rosmarinic Acid (µg/g d.w.)	The Sum of Phenolic Acids (µg/g d.w.)	The Sum of Phenolic Compounds (µg/g d.w.)
1	20	60	5	206.59 ± 0.28	1053.93 ± 1.41	1091.82 ± 1.48
2	100	60	5	18.19 ± 0.06	18.19 ± 0.06	24.63 ± 0.09
3	20	60	45	238.12 ± 0.32	1177.45 ± 1.56	1224.58 ± 1.64
4	100	60	45	149.26 ± 0.21	330.65 ± 0.50	383.61 ± 0.60
5	20	20	25	254.64 ± 0.34	1142.60 ± 1.50	1185.53 ± 1.58
6	100	20	25	8.26 ± 0.03	8.26 ± 0.03	8.44 ± 0.03
7	20	100	25	61.24 ± 0.11	570.75 ± 0.78	580.83 ± 0.80
8	100	100	25	33.92 ± 0.07	92.39 ± 0.20	142.17 ± 0.29
9	60	20	5	317.40 ± 0.41	1302.98 ± 1.69	1358.20 ± 1.78
10	60	20	45	253.07 ± 0.34	1108.33 ± 1.47	1149.89 ± 1.55
11	60	100	5	412.44 ± 0.53	1396.61 ± 1.82	1459.35 ± 1.93
12	60	100	45	142.60 ± 0.21	863.03 ± 1.17	909.48 ± 1.26
13	60	60	25	420.13 ± 0.46	1458.90 ± 1.87	1559.45 ± 2.04
14	60	60	25	406.35 ± 0.53	1419.25 ± 1.56	1522.60 ± 2.03
15	60	60	25	415.72 ± 0.51	1437.80 ± 1.23	1545.94 ± 1.94

**Table 3:** Regression coefficients and analysis of variance (ANOVA) of the model for the response variables for *Ocimum basilicum*.

	Y_1_		Y_2_		Y_3_	
Regression Coefficients	Rosmarinic Acid (µg/g d.w.)		The Sum of Phenolic Acids (µg/g d.w.)		The Sum of Phenolic Compounds (µg/g d.w.)	
	Effect	*p*-Value	Effect	*p*-Value	Effect	*p*-Value
β_0_	254.74	0.0004 *	755.43	0.0001 *	793.21	0.0000 *
Linear						
β_1_	−226.90	0.0035 *	−851.32	0.0003 *	−856.61	0.0003 *
β_2_	−75.48	0.0305 *	−187.85	0.0061 *	−180.20	0.0059 *
β_3_	1.66	0.9133	23.95	0.2467	37.55	0.1140
Crossed						
β_12_	149.79	0.0143 *	327.99	0.0036 *	369.21	0.0025 *
β_13_	7.74	0.7108	94.47	0.0414 *	113.11	0.0261 *
β_23_	−134.62	0.0176 *	−169.47	0.0134 *	−170.78	0.0117 *
Quadratic						
β_11_	267.63	0.0012 *	753.92	0.0002 *	800.74	0.0001 *
β_22_	66.23	0.0197 *	231.24	0.0020 *	262.67	0.0014 *
β_33_	−22.82	0.1365	39.68	0.0615	60.76	0.0246 *
R^2^	0.9855		0.9980		0.9967	
*p*-model	0.0010 *		0.0000 *		0.0000 *	
*p* lack of fit	0.3525		0.2467		0.1140	

* Significant at α ≤ 0.05

**Table 4:** Optimized conditions for UAE via a sonotrode for *Ocimum basilicum* leaves.

	Optimal Conditions		
Ethanol/water (*v*/*v*)	50		
Amplitude (%)	50		
Time (min)	5		
	**Rosmarinic acid**	**Sum of phenolic acids**	**Sum of phenolic compounds**
Predicted value (µg/g d.w.)	529.97 ± 59.37	1657.15 ± 86.09	1749.04 ± 80.91
Obtained value (µg/g d.w.)	531.00 ± 2.65	1695.33 ± 5.03	1762.00 ± 19.29
CV (%)	0.14	1.61	0.52

## Data Availability

Not applicable.
